# Perception of drug teratogenicity among general practitioners and specialists in obstetrics/gynecology: a regional and national questionnaire-based survey

**DOI:** 10.1186/s12884-016-1025-6

**Published:** 2016-08-17

**Authors:** Charlotte Gils, Anton Pottegård, Zandra Nymand Ennis, Per Damkier

**Affiliations:** 1 Department of Clinical Biochemistry & Pharmacology, Odense University Hospital, Odense, Denmark; 2 Clinical Pharmacology and Pharmaceutics, Department of Public Health, University of Southern Denmark, Odense, Denmark

**Keywords:** Drugs, Pregnancy, Teratogenicity, Risk, Perception

## Abstract

**Background:**

Estimating the true risk of fetal malformations attributable to the use of medications is difficult and perception of risk by health professionals will impact their counseling and treatment of patients who need medication during pregnancy. The objective of this study was to assess the perception of the teratogenic risk of 9 commonly and 3 rarely prescribed drugs among general practitioners and specialists in obstetrics/gynecology.

**Methods:**

All 811 general practitioners in the Region of Southern Denmark and all 502 specialist obstetricians/gynecologists in Denmark as a whole were invited to participate in the study based on an online questionnaire. Medians and interpercentile ranges of the perceived background risk and perceived risks for each of the drugs were included in the questionnaire.

**Results:**

One hundred forty three (18 %) general practitioners and 138 (27 %) obstetricians/gynecologists participated. Estimates provided by the participants were generally in accordance with current knowledge of drugs with established safety during pregnancy. Perceptions of risks associated with warfarin and retinoid exposure were severely underestimated.

**Conclusions:**

Understanding of teratogenic background risk and specific risks associated with in utero exposure to 12 different drugs generally approached the established knowledge. The risk associated with warfarin and retinoid exposure was severely underestimated by both groups of health care professionals, while general practitioners specifically overestimated the risk of sertraline and citalopram to some extent. In Denmark, general practitioners can prescribe antidepressants, and even minor misconceptions of the teratogenic potential of citalopram and sertraline may be of clinical relevance. In Denmark, systemic retinoids can only be prescribed by a dermatologist, and warfarin treatment is only rarely initiated in women of the fertile age without involvement of specialists in internal medicine. Hence, the active knowledge on the teratogenic potential of these drugs is likely to be less accurate among general practitioners and obstetricians/gynecologists; although still of clinical importance since these specialists are largely involved in the counselling of pregnant women.

**Electronic supplementary material:**

The online version of this article (doi:10.1186/s12884-016-1025-6) contains supplementary material, which is available to authorized users.

## Background

Knowledge about the risk of medications being teratogenic became apparent after the thalidomide disaster [1, 2] some 50 years ago. The tragedy gave rise to concerns on the safety of drugs during pregnancy and prompted international agencies to develop systematic preclinical reproductive testing protocols [2]. Estimating the true risk of fetal malformations attributable to the use of medications is difficult and controversial. While randomized controlled trials are seen as the gold standard for assessing safety and efficacy of medications, pregnant women are routinely excluded from such trials [3]. This places a heavy reliance on observational studies and pharmacoepidemiological data to provide evidence in support of informed decision making on medication use during pregnancy [3]. Perception of risk by health professionals will impact their level of counseling and treatment of patients who need medication during pregnancy. Overestimating this risk can lead to insufficient treatment of patients, whereas underestimating may lead to hazardous practice. Legal medical issues may additionally complicate matters, and in case of *Bendectin* such issue led to the market withdrawal of a documented safe and effective product [4]. Perceptions of risk by patients impact their decision on whether or not to use medicine during pregnancy, and the risk perceived by patients has been shown to be heavily dependent on the information received from their physician [5–7]. In recent surveys, 77 % of women stated that they needed information about drug use during their pregnancy, and 62 % of women believed it would be better for the fetus if they refrained from using drugs that they would otherwise have used if not pregnant [8, 9]. Teratogenic risk perception among health care professionals has only been subject to small-scale studies mostly pertaining to general practitioners (GP) while only about 200 obstetricians/gynecologists (OB/GYN) have been subject to such study on teratogenic drug risk-perception on a world-wide scale [10–15].

## Aim of the study

The purpose of this study was to investigate the perception of teratogenic risk of nine different commonly and three rarely prescribed drugs among all GP in the Southern Region of Denmark and all OB/GYN in Denmark.

## Methods

### Study population

We included two groups of health professionals: All GP (*n* = 811) in the Region of Southern Denmark (approximately 22 % of the Danish population) and all OB/GYN holding a specialist authorization in Denmark (*n* = 502). The demographic characteristics, patterns of health care utilization and medication use are very homogenous in Denmark, and the Region of Southern Denmark compares well to other regions [16]. Adherence to RATS guideline is documented in Additional file 1.

### Data collection

Information was gathered by anonymous self-completed questionnaires. The questionnaire was developed through *SurveyXact* and was made available at a website for internet surveys (https://www.survey-xact.dk). An invitation to the study, including a link and a code to the questionnaire, were sent to the study participants by mail. Email addresses could not be obtained as the respective organizations declined to release email addresses for the purpose of this study. The online questionnaire was accessible from November 19, 2012 to February 28, 2013. Translations of the cover letter to participating physicians and the questionnaire are provided in Additional file 2. 

### Measurements of risk perception

The participants were asked to estimate the overall risk of malformations in the background population. To evaluate the perception of the teratogenic risk of specific medications during pregnancy, the participants were asked to give their best estimate based on their active knowledge. Estimates were to be entered as an integer between 0 and 100 %, without using references of any kind while spending less than 5 min to complete the survey. The survey comprised drugs with documented no or minor increased risks of teratogenicity (phenoxymethylpenicillin, metoclopramide, citalopram and sertraline representing selective serotonin reuptake inhibitors (SSRIs), benzodiazepines, inhalation glucocorticoids, fluconazole, and lamotrigine) [17–19], one drug with insufficient data for risk estimation (quetiapine) [20] and drugs with documented increased risk (retinoids, warfarin and thalidomide) [17–19]. The “minor” risk designation is covering some discrepancies and controversy on risk estimation with respect to SSRI exposure and risk of cardiovascular malformations. No more than a 1.71-fold (the upper bound of the 95 % CI) increased risk of cardiovascular malformations and no more than a 1.26-fold (the upper bound of the 95 % CI) increased risk of overall major malformations, using the most comprehensive data from meta-analysis data [21] and a recent study comprising all Nordic data available appears likely [22].

For all drugs, the most common trade names were stated in the questionnaire together with the generic names.

### Data analysis

The questionnaires were analyzed by standard non-parametric descriptive statistics, using STATA release 12.0 (StataCorp, College Station, TX, USA). The Wilcoxon Rank-Sum Test was used for inferential statistics comparing the responses between GP and OB/GYN.

## Results

One hundred forth three (18 %) GP and 138 (27 %) OB/GYN answered the questionnaire. Responses from 5 GP were excluded as they had entered baseline malformation rates of 100 % or higher. Among the remaining GP, 28 had answered less than half the questions, 24 had answered more than half but not all questions and 91 had answered all questions. The corresponding values among OB/GYN were 29, 34 and 75. Sensitivity analysis with respect to numbers of questions answered did not alter the results (data not shown). A substantial proportion of responders had entered values for risks associated with specific drug exposure as excess risk (relative to background risk) rather than absolute risks. In these cases, values entered for teratogenic risks associated with specific drug exposures were lower than the values entered for the overall background risk. In such cases, we added the risk value entered for risk associated with the specific drug exposure to the value entered for the overall background risk. We corrected the values for 84 (59 %) of GP and 63 (46 %) of OB/GYN.

**Fig. 1:**
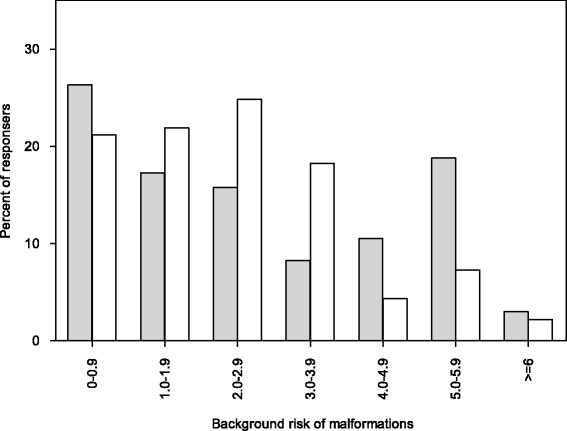
Distribution of perceived background risk. Distribution of GPs (*grey*) and OB/GYN (*white*) perception of the overall risk of congenital malformations in the background population

**Fig. 2:**
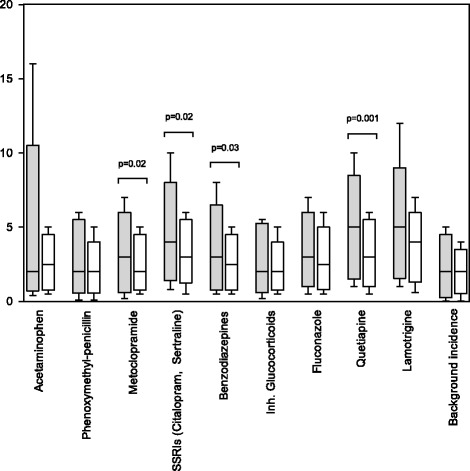
Perception of background risk and for drugs with no or minor excess risk. Perception of teratogenic risk by GPs (*grey*) and OB/GYN (*white*) of drugs known to be safe during pregnancy and the overall background incidence. Boxplot with medians and 50 % interpercentil ranges with whiskers from the 10th to 90th percentiles. Statistically significant *p*- values are indicated

**Fig. 3:**
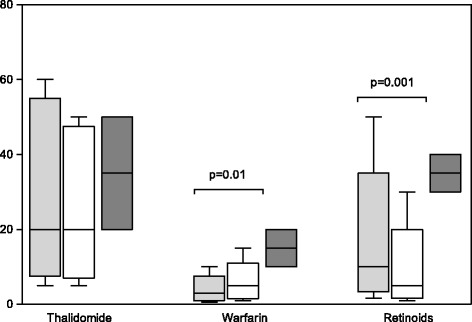
Perceptions of risk for known teratogenic drugs. Perception of teratogenic risk by GPs (*grey*) and OB/GYN (*white*) of drugs with documented teratogenic potential. Boxplot with medians and 50 % interpercentil ranges with whiskers from the 10th to 90th percentiles representing the answers from GPs (grey and OB/GYN (*white*). The black boxes represent the estimates of “true” malformation risks for each of the drugs. Statistically significant *p*-values are indicated

The results are presented in Table 1 and illustrated in Figs. 1, 2 and 3. Generally, the median values of risk perception indicate a realistic perception of drugs with established safety. Notably, both GP and OB/GYN had perceptions of teratogenic potential for retinoids and warfarin that were substantially below the risk documented in the literature [17, 18]. The distribution of percentiles demonstrated a greater variability in responses for the OB/GYN population compared to the GP responses. Statistically different perceptions of teratogenic potential were found for metoclopramide, citalopram/sertraline, benzodiazepines, quetiapine, warfarin and retinoids with GP generally giving higher risk estimates. In terms of absolute differences, median values were small with the largest differences being for retinoids, warfarin and citalopram/sertraline.

**Table 1:** Perception of teratogenicity among GP and OB/GYN responders according to drug

Drug	GP responders	OB/GYN responders	*p*-value^a^
	Median & [50 %] and 90 % IPR	Median & [50 %] 90 % IPR	
Acetaminophen	2.0 [1.0–5.0]; 0.4–16	2.5 [1.0–4.0]; 0.5–5.0	0.41
Phenoxymethylpenicillin	2.0 [1.0–5.0]; 0.1–6.0	2.0 [1.0–3.0]; 0,1–5,0	0.29
Metoclopramide	3.0 [1.0–5.0]; 0.20–7.0	2.0 [1.0–4.0]; 0.5–5.0	0.02
Citalopram/sertraline	4.0 [2.0–6.0]; 0.8–10.0	3.0 [2.0–5.0]; 0.5–6.0	0.02
Benzodiazepines	3.0 [1.0–5.0]; 0.5–8.0	2.5 [1.0–4.0]; 0.5–5.0	0.03
Inhaled glucocorticoids	3.0 [1.0–5.0]; 0.2–5.5	2.0 [1.0–3.0]; 0.5–5.0	0.54
Fluconazole	3.0 [1.5–5.0]; 0.5–7.0	2.5 [1.1–4.0]; 0.5–6.0	0.45
Quetiapine	5.0 [2.0–7.0]; 1.0–10	3.0 [1.5–5.0]; 0.5–6.0	0.001
Lamotrigine	5.0 [2.1–6.0]; 1.0–12	4.0 [2.0–5.0]; 0.6–7.0	0.25
Thalidomide	20 [10–50]; 5.0–60	20 [9.0–45]; 5.0–50	0.14
Warfarin	3.0 [1.5–5.0]; 0.5–10	5.0 [2.1–7.0]; 1.0–15	0.01
Retinoids	10 [5.0–20]; 1.6–50	5.0 [2.1–10]; 1.0–30	0.001
Background incidence	2.0 [0.5–4.0]; 0.02–5.0	2.0 [1.0–3.0]; 0.03–4.0	0.36

[50 %] and 90 % IPR: 50 % and 90 % interpercentil ranges. ^a^: Wilcoxon signed rank sum test

## Discussion

This is one of the largest systematic surveys on risk perception following in utero exposure to drugs among health care professionals reported in the literature, and the only study reporting on a nation-wide basis. Overall, the responses were quite comparable with both between GP and OB/GYN responders and compared to literature data, though the variation in risk perception appeared somewhat larger among GP. Some statistically significant differences for individual drugs materialized, as GP tended to slightly overestimate the risk associated with exposure to citalopram/sertraline, metoclopramide and benzodiazepines. Both GP and OB/GYN appeared to overestimate risk associated with lamotrigine. Lastly, we found a substantial underestimation between the perceived risk for retinoids and warfarin and the risk documented in the literature among both GP and OB/GYN responders.

Generally, our results demonstrate a better understanding of teratogenic potential that otherwise reported in the literature. In a Spanish study from 2001, perception of malformation rates following in utero exposure were acetaminophen (5 %), amoxicillin (6 %), metoclopramide (14 %), benzodiazepines (10 %), warfarin (53 %), etretinate (96 %), and thalidomide (82 %) among 104 physicians [10]. Damase-Michel et al. found perception of malformation rates for acetaminophen (20 %), amoxicillin (14 %), metoclopramide (37 %), bromazepam (28 %), warfarin (59 %), isotretionin (89 %), and thalidomide (92 %) among 103 GP in a French region [11]. Contrary to these rather substantial overestimations, 74 Norwegian GP, assigned low values (below 1.5 using a visual analogue scale from 0 to 10) for teratogenic risk perception for metoclopramide, acetaminophen and pivmecillinam, while escitalopram was rated 3.7 [12]. In a recent Brazilian study, more than 50 % of GP and obstetricians asked (total *n* = 80) had a perceived risk of congenital malformations of more than 5 % associated to first-trimester exposure to antidepressants, antipsychotics, benzodiazepine and anticonvulsants [13]. Serious overestimation of teratogenic risks associated with radiography and CT-scan during early pregnancy by GP and OB/GYN has also been reported [14]. The overall reasonable risk estimates from the responders in our study may to some extent be attributed to selection bias (see study limitations below), but dissemination of relevant information in a systematic and useable form is likely a contributing factor [23–25]. The by far most commonly used Danish drug information resource by health care professionals was completely restructured in 2005 with respect to pregnancy and lactation recommendations [19]. This Danish online analogue to the Physicians’ Desk Reference has since 2005 implemented a transparent and uniform algorithm, which provides the clinician with information on the level of scientific evidence; risk estimations and a clinical recommendation (see Additional file 3 for a translation of these principles).

The difference in perception of the teratogenic potential for citalopram and sertraline between GP and OB/GYN is noteworthy for several reasons: The issue of SSRIs during pregnancy has been subject to an extensive coverage within the health care community [21–32]. Numerous data are available and a recent comprehensive meta-analysis documented more than 56,000 exposed pregnancies [21]. This analysis found no overall increased risk of major congenital malformations (OR 1.07, CI 0.99–1.17), while some excess risk (OR 1.36, CI: 1.08–1.71) appears likely with respect to cardiac malformations. A complete analysis from register data in all Nordic countries comprising almost 37000 exposed pregnancies found largely comparable estimates. The overall major malformation risk was slightly increased (OR 1.13, CI: 1.06–1.20) and the risk of cardiovascular malformations was similar (OR 1.15, CI 1.05–1.17) [22]. Interestingly, the latter signal disappeared completely in a subsequent sibling-controlled analysis. Another recent case-control study based on EUROCAT register data found a comparable level of association for overall cardiovascular risk (OR 1.41, CI 1.07–1.86) with no apparent specific signals for paroxetine and fluoxetine [29]. The EUROCAT data were not subject to any covariate control analysis, and may be subject to reporting and observer bias as these data are typically only reported from certain regions in participating countries. This is quite different from the Nordic dataset, which represents a complete cohort of all exposed pregnancies in the Nordic countries [22]. It should be noted that there is some overlap of data among these studies, as data from the Nordic countries to a varying degree contribute to all three analyses. While overall no clinically important risk of major congenital malformations following first-trimester in utero exposure to SSRIs has materialized from the best meta-analyses available, a slightly increased relative risk of cardiovascular malformations appears consistently reproduced. One study suggests that confounding by indication be an issue as identical signals were found for pregnant women pausing SSRI treatment during pregnancy [30]. Ascertainment bias may contribute to these observations as well [31]. The clinical significance of this increase in absolute risk of cardiovascular malformations is subject to much controversy, though most do not believe it to preclude medical treatment [32–34]. Treating physicians are sometimes poorly helped by regulatory information that often leads to confusion: At the time this survey was performed, different pregnancy labeling existed for generic variations of citalopram in Denmark. The Summary of Product Characteristics (SmPC) from the original marketing authorization holder stated “…more than 2500 exposed pregnancies does not indicate excess risk of unwanted pregnancy outcome, but citalopram must not be used during pregnancy unless an absolute necessity” [35]. SPC for a generic product simply stated”… should not be used during pregnancy as data are insufficient…” [36]. None of these adhered to the guideline on pregnancy labeling given the data at hand [37]. This discrepancy has later been corrected by the Danish Health and Medicines Authority. The perception of teratogenic potential has a direct influence on patient compliance, and misconceptions may lead to unwarranted discontinuation of antidepressant treatment [38, 39]. Thus, even minor misconceptions of the teratogenic potential of citalopram and sertraline may be of clinical relevance.

The risks of congenital malformations associated with exposure to warfarin and retinoids were substantially underestimated by GP and OB/GYN responders. This is in contrast to other studies in which these same risks were grossly overestimated [10, 11]. In Denmark, systemic retinoids can only be prescribed by a dermatologist, and warfarin treatment is rarely initiated in women of the fertile age without involvement of specialists in internal medicine (deep venous thrombosis) or cardiologists (heart valve replacement) [40]. Hence, the active knowledge on teratogenic potential of these drugs is likely to be less accurate among GP and OB/GYN.

Lamotrigine is considered safe during pregnancy, except at very high doses, as substantial amounts of data do not suggest an increased risk of unwanted fetal effects [41]. Responders in our study assigned risks of about 4–5 % for congenital malformations, with similar perception of teratogenicity among GP and OB/GYN. Lamotrigine treatment is primarily initiated by specialists within psychiatry or neurology, and the active knowledge on lamotrigine's teratogenic potential among other healthcare professionals may not be adequate. Anticonvulsants have generally been associated with teratogenic potential, especially carbamazepine, valproate and phenytoin [41, 42]. The knowledge thereof may have had a spillover effect on the perception of the teratogenic potential of lamotrigine among GP and OB/GYN. The clinical importance of this is mitigated by the same circumstances, as physicians’ advice to pregnant women receiving lamotrigine, would likely include consulting the specialist responsible for initiation of the treatment.

This study comes with a number of limitations. While the absolute number of responders is high compared to most other relevant studies, the response rates in our questionnaire are unimpressive but comparable to those of another recent study [15]. We also have no way of determining whether in fact responders only used actual knowledge or made use of reference sources while filling out the questionnaire. Bias in both cases would likely be conservative as invitees may be more inclined to respond if they feel that their actual knowledge on the subject would be clinically sufficient. We were unable to analyze results from responders and non-responders according to demographic characteristics of interest such as age, sex, or length of medical experience. Accordingly, generalization of our results should be made with caution.

## Conclusion

In conclusion, responders to this survey demonstrated an understanding of teratogenic background risk and specific risks associated with in utero exposure to 12 different drugs that generally approached established knowledge. The risks associated with warfarin and retinoid exposure were severely underestimated by both groups of health care professionals, while GP specifically overestimated the risk of sertraline and citalopram.

## Additional files

Additional file 1: RATS guideline adherence. (DOCX 16 kb) Additional file 2: Cover letter to participants and online questionnaire. (DOC 27 kb) Additional file 3: Classification of medication use during pregnancy from the Danish PDR. (DOCX 17 kb)
