# Supplementary material for: Perception of drug teratogenicity among general practitioners and specialists in obstetrics/gynecology: a regional and national questionnaire-based survey
Source: BMC Pregnancy Childbirth. 2016 Aug 17;16:226. doi: 10.1186/s12884-016-1025-6 (PMC4988043; doi:10.1186/s12884-016-1025-6)
Supplement: Additional file 1: — RATS guideline adherence. (DOCX 16 kb) [file 12884_2016_1025_MOESM1_ESM.docx]

**Additional file 1**

**RATS Guideline adherence**

### Research question explicitly stated:

*This is explicitly stated in the end of the introduction*

### Research question justified and linked to the existing knowledge base (empirical research, theory, policy):

The research question is justified and linked to the existing knowledge base in the introduction

### Study design described and justified i.e., why was a particular method (e.g., interviews) chosen:

This is specified in the methods section

### Criteria for selecting the study sample justified and explained:

This is explained in the introduction

### Details of how recruitment was conducted and by whom

This is explained in the data collection section in the Methods

### Details of who chose not to participate and why

This has been discussed in the section on limitations by the end of the discussion

### Method(s) outlined and examples given (e.g., interview questions)

The questionnaire is submitted as Supplementary Material II

### Study group and setting clearly described

Described in the Methods section

### End of data collection justified and described

The online questionnaire was accessible from 19th of November 2012 to 28th of February 2013. The end of data collection was prospectively determined by the authors as a subjective assessment when it was believed that no more respondents would answer the questionnaire.

### Do the researchers occupy dual roles (clinician and researcher)? Are the ethics of this discussed? Do the researcher(s) critically examine their own influence on the formulation of the research question, data collection, and interpretation?

The researchers do not occupy dual roles. Ethics is accounted for.

### Informed consent process explicitly and clearly detailed

Not applicable.

### Anonymity and confidentiality discussed

This is explained in the beginning of the Methods

### Ethics approval cited

This study was a completely anonymous questionnaire not involving person-specific health-care related information. The Danish law does not require ethical approval in such cases. The study was approved by the Danish Data Protection Agency.

### Analytic approach described in depth and justified

Described in data analysis

### Discussion and interpretation

Results have been discussed and put in perspective to results from other similar studies. The results have been discussed in a health perspective as related to the academic challenges and controversies within the field.
