# Supplementary material for: Perception of drug teratogenicity among general practitioners and specialists in obstetrics/gynecology: a regional and national questionnaire-based survey
Source: BMC Pregnancy Childbirth. 2016 Aug 17;16:226. doi: 10.1186/s12884-016-1025-6 (PMC4988043; doi:10.1186/s12884-016-1025-6)
Supplement: Additional file 3: — Classification of medication use during pregnancy from the Danish PDR. (DOCX 17 kb) [file 12884_2016_1025_MOESM3_ESM.docx]

**Additional file 3**

**Classification of medication use during pregnancy from the Danish PDR**

**Translated from Danish:** [**http://pro.medicin.dk/Specielleemner/Emner/315229**](http://pro.medicin.dk/Specielleemner/Emner/315229) **and** [**http://pro.medicin.dk/Specielleemner/Emner/317943**](http://pro.medicin.dk/Specielleemner/Emner/317943) **, accessed December 28^th^, 2015**

Medication used during pregnancy is classified in five categories with a short accompanying text. This is a decision support to the treating physician in order to qualify the effect of the drugs in relation to possible risks to the pregnant woman or foetus. The classification cannot be used indiscriminately - included drugs classified as "may be used if necessary" - without such considerations of pros and cons, and in principle the lowest dose for the shortest duration of time should always be used.

**May be used if necessary**

- There is no evidence of harmful foetal effects
- Data exists for at least 700 1. trimester exposed pregnant women without indication of higher frequency of harmful effect to the foetus

*Or*

- The systemic absorption is so insignificant that harmful effect to the foetus is judged unlikely

*Or*

- The medication is a physiological substance which is not considered to have any harmful effect to the foetus

**Should not be used, insufficient data**

- There is no evidence of harmful effect to the foetus
- Data exists for less than 700 1. trimester exposed pregnant women without indication of increased risk of harmful effect to the foetus

**Should only be used in certain circumstances**

- The specific indications for use of this medication may mitigate the lack of evidence for safety or likely harmful effect to the foetus. This pertain for instance to some antiepileptic medications, warfarin and lithium.
- Restrictions may apply: dose, duration, timing according to gestational age of pregnancy

**Cannot be used**

- There is evidence of harmful effect to the foetus
- The indication of use of the medication implies that it will never be necessary to use in pregnant women

**Not relevant**

- Any recommendation is considered irrelevant in terms of the indication of the medication

**Data sources accessed**

The foundation for assembling the quantitative estimates for the decision algorithm above the following literature is regularly consulted for each drug in question. In some select cases additional sources are consulted:

[**http://www.ncbi.nlm.nih.gov/pubmed**](http://www.ncbi.nlm.nih.gov/pubmed)

- Free text string search using "generic drug name" AND "pregnancy"

**Briggs GG. Drugs in pregnancy and lactation 9. edition, Lippincott Williams & Wilkins 2011.**

**Micromedex.com**

**janusinfo.se**

**Currently approved Summary of Product Characteristics, SmPC**
