# Supplementary material for: Perception of drug teratogenicity among general practitioners and specialists in obstetrics/gynecology: a regional and national questionnaire-based survey
Source: BMC Pregnancy Childbirth. 2016 Aug 17;16:226. doi: 10.1186/s12884-016-1025-6 (PMC4988043; doi:10.1186/s12884-016-1025-6)
Supplement: Additional file 2: — Cover letter to participants and online questionnaire. (DOC 27 kb) [file 12884_2016_1025_MOESM2_ESM.doc]

**Additional file 2: Cover letter to participants and online questionnaire.**

**Cover Letter**

Five minutes of your time: Pregnancy and drugs; a scientific study.

Dear Colleague

We are performing a scientific study on the perception of teratogenic potential of drugs used during pregnancy among health care professionals. We hope that you are able and willing to use five minutes to complete an online questionnaire:

- Please use active knowledge/intuition only – do not use any references
- All answers are handled anonymously

To access the questionnaire enter [www.datafabrikken.dk](http://www.datafabrikken.dk/) in your browser. Enter the following code:

XXXXXXX.

Thank you for your participation.

**Online Questionnaire:**

Pregnancy and drugs

When completing the questionnaire it is important to answer on the basis of active knowledge or intuition using a numeric value without decimal digits. Please refrain from the use references of any kind. The responses are anonymous.

Completion of the questionnaire will take about 5 minutes.

Thank you for your participation.

1. What is the background risk (in percent) of congenital malformations in the Danish population?
2. How many percent of children, exposed to acetaminophen (Pamol®, Panodil® Pinex®), during pregnancy, are born with malformations?
3. How many percent of children, exposed to phenoxymethylpenicillin (Vepicombin®, Primcillin®) during pregnancy, are born with malformations?
4. How many percent of children, exposed to metoclopramide (Emperal®, Primperan®) during pregnancy, are born with malformations?
5. How many percent of children, exposed to selective serotonin reuptake inhibitors (Citalopram, Sertralin) during pregnancy, are born with malformations?
6. How many percent of children, exposed to benzodiazepines (Diazepam, Oxazepam) during pregnancy, are born with malformations?
7. How many percent of children, exposed to inhalation glucocorticoids (Spirocort®, Flixotide®) during pregnancy, are born with malformations?
8. How many percent of children, exposed to fluconazole (Diflucan®) during pregnancy, are born with malformations?
9. How many percent of children, exposed to quetiapine (Seroquel®, Alzen®) during pregnancy, are born with malformations?
10. How many percent of children, exposed to lamotrigine (Lamictal®) during pregnancy, are born with malformations?
11. How many percent of children, exposed to warfarin (Marevan®) during pregnancy, are born with malformations?
12. How many percent of children, exposed to retinoids (Neotigason®) during pregnancy, are born with malformations?
13. How many percent of children, exposed to thalidomide during pregnancy, were born with malformations?
